# Supplementary material for: Results of DUET: A Web-Based Weight Loss Randomized Controlled Feasibility Trial among Cancer Survivors and Their Chosen Partners
Source: Cancers (Basel). 2023 Mar 3;15(5):1577. doi: 10.3390/cancers15051577 (PMC10000640; doi:10.3390/cancers15051577)
Supplement: Supplementary file 1 [file cancers-15-01577-s001.zip › cancers-2192383-supplementary.pdf]

Table S1. Self-reported quality-of-life and factors associated with behavior change among participants in wait-listed vs. DUET study arms at baseline, and 3- and 6-month follow-up.

	Waitlist Control			DUET Intervention			Significance (p-values)		
	Baseline Mean (SD)	3M Mean (SD)	6M Mean (SD)	Baseline Mean (SD)	3M Mean (SD)	6M Mean (SD)	Between Arm	Within Arm	Time x Arm
Quality of Life (PROMIS)									
Physical Health									
Survivors	43.4 (5.7)	44.0 (6.8)	46.2 (5.6)*	45.5 (6.6)	44.8 (5.7)	47.3 (7.1)*	0.290	**0.025**	0.798
Partners	51.1 (9.1)	51.5 (9.3)	51.3 (9.7)	44.4 (5.6)	44.1 (5.2)	44.7 (5.4)	0.579	0.288	0.096
Dyads	43.9 (5.2)	44.4 (6.2)	44.4 (5.6)	44.9 (6.1)	44.5 (5.4)	46.0 (6.4)	0.280	0.387	0.553
Mental Health									
Survivors	48.6 (8.2)	49.6 (8.1)	50.4 (9.1)	48.4 (5.7)	47.3 (7.5)	48.7 (8.5)	0.529	0.221	0.385
Partners	50.0 (6.6)	49.5 (8.5)	50.9 (7.9)	51.1 (9.1)	51.5 (9.3)	51.3 (9.7)	0.674	0.413	0.627
Dyads	49.2 (7.6)	49.5 (8.4)	50.6 (8.7)	49.8 (8.3)	49.3 (8.6)	50.0 (9.1)	0.901	0.079	0.441
Self-efficacy (% confidence [SD])									
Low Calorie diet									
Survivors	69.7(19.0)	69.4(24.0)	70.0(22.8)	64.5(20.3)	61.8(20.0)	70.7(14.3)	0.415	0.154	0.297
Partners	73.5(23.8)	74.3(24.7)	71.0(21.4)	66.8(23.4)	73.2(19.3)	75.9(19.1)	0.769	0.368	0.158
Dyads	71.5(21.6)	72.7(24.3)	70.9(22.2)	65.7(21.8)	67.4(20.3)	73.3(17.0)	0.346	0.127	0.080
Increased PA									
Survivors	49.9(25.6)	49.3(29.8)	45.1(26.3)	49.6(26.0)	45.8(22.8)	55.7(21.8)	0.704	0.684	0.096
Partners	51.3(29.2)	46.0(24.6)	48.0(23.5)	44.7(24.6)	51.1(30.3)	47.8(26.1)	0.834	0.990	0.362
Dyads	51.4(27.3)	48.9(27.0)	48.2(24.0)	47.1(25.2)	48.3(26.6)	51.7(24.2)	0.820	0.746	0.389
Social Support (% of items support reported as "often" or "everyday")									
Low Calorie diet									
Survivors	53.8(37.2)	51.0(39.2)	51.0(43.3)	43.8(37.7)	51.8(40.8)	42.9(37.2)	0.471	0.664	0.665
Partners	41.7(36.6)	46.9(39.9)	40.2(31.7)	45.5(39.7)	61.5(37.6)	55.4(41.6)	0.237	0.202	0.602
Dyads	47.9(37.5)	50.5(39.2)	46.3(38.3)	44.6(38.4)	56.5(39.2)	49.1(39.6)	0.866	0.160	0.601
Increased PA									
Survivors	45.2(37.4)	46.0(40.0)	43.3(43.9)	45.5(40.9)	43.8(38.3)	49.1(37.6)	0.911	0.987	0.732
Partners	39.6(39.0)	43.8(41.9)	38.0(36.8)	34.8(33.6)	50.0(38.7)	42.9(35.9)	0.864	0.234	0.604
Dyads	43.8(38.1)	46.8(40.2)	42.0(40.7)	40.2(37.4)	46.8(38.3)	46.0(36.5)	0.923	0.491	0.698
Barriers (% of items reported barriers)									
Low Calorie diet									
Survivors	24.2(25.3)	21.2(19.2)	18.8(21.4)†	25.7(19.3)	20.7(19.0)	11.1(12.9)†‡	0.627	**<0.001**	0.168
Partners	13.3(14.3)	20.0(25.0)	14.3(20.2)	17.9(20.2)	10.8(15.2)	10.4(12.0)	0.491	0.385	0.051
Dyads	19.2(21.7)	20.2(22.3)	15.5(19.7)¶	21.8(20.0)	15.9(17.9)§	10.7(12.3)¶	0.517	**<0.001**	0.106
Increased PA									
Survivors	27.5(23.2)	26.5(20.2)	27.8(26.4)	22.3(16.4)	21.3(20.5)	13.4(14.4)	0.091	0.207	0.130
Partners	18.1(15.5)	14.9(12.7)‖	13.7(14.1)#	26.3(20.2)	15.4(15.4)‖**	17.0(18.2)#††	0.266	**0.001**	0.217
Dyads	22.1(20.2)	20.2(19.6)	20.2(22.2)‡‡	24.3(18.4)	18.4(18.3)	15.2(16.3)‡‡,‡	0.647	**0.003**	0.138

*Post-hoc analyses indicate that the 6M values are significantly greater than 3M values (p=0.044).
†Post-hoc analyses indicate that the 6M values are significantly lower than baseline (p<0.001).
‡Post-hoc analyses indicate that the 6M value is significantly lower than baseline (p=0.001).
¶ Post-hoc analyses indicate that the 6M values are significantly lower than the 3M values (p-0.023).
§ Post-hoc analyses indicate that the 3M value is significantly lower than baseline (p<0.001).
‖ Post-hoc analyses indicate that the 3M values are significantly lower than baseline (p=0.005).
#Post-hoc analyses indicate that the 6M values are significantly lower than baseline (p=0.003).
**Post-hoc analyses indicate that the 3M value is significantly lower than baseline (p=0.006).
††Post-hoc analyses indicate that the 6M value is significantly lower than baseline (p=0.017).
‡‡Post-hoc analyses indicate that the 6M values are significantly lower than baseline (p=0.002).
